# Erratum: Nováková et al. An Analysis of Food Waste in Czech Households—A Contribution to the International Reporting Effort. *Foods* **2021**, *10*, 875

**DOI:** 10.3390/foods10061191

**Published:** 2021-05-25

**Authors:** Petra Nováková, Tomáš Hák, Svatava Janoušková

**Affiliations:** 1 Faculty of Humanities, Charles University, 110 00 Prague, Czech Republic; novakova.petra.cz@gmail.com; 2 Environment Centre, Charles University, 110 00 Prague, Czech Republic; svatava.janouskova@czp.cuni.cz; 3 Faculty of Science, Charles University, 110 00 Prague, Czech Republic

The *Foods* Editorial Office would like to update the error in the original published version [1]. The Table 1 will be updated to a colored background. Each color meaning is explained in table footer as well as in the text of the article. 

We would like to apologize for any inconvenience caused to the authors and readers by this error. We will update the article, and the original version will remain available on the article webpage. 

## Figures and Tables

**Table 1:** Accuracy and reliability (*** high, ** medium) of the food waste measurement methods suggested by the European Commission (adapted based on [46]).

Stage of the FCS	Methods for Food Waste Measurement					
Primary production	Direct measurement (weighing or volumetric) ***	Questionnaires and interviews **	Mass balance **		Coefficients and production statistics **	
Processing and manufacturing						
Retail and food distribution				Waste composition analysis ***	Counting/ scanning **	
Restaurants and food services						Diaries **
Households						

Direct methods in the green-shaded cells; indirect methods in the blue-shaded cells; and yellow-shaded cells include both direct and indirect methods.
